# IGFBP6 controls the expansion of chemoresistant glioblastoma through paracrine IGF2/IGF-1R signaling

**DOI:** 10.1186/s12964-018-0273-7

**Published:** 2018-09-19

**Authors:** Claudia R. Oliva, Brian Halloran, Anita B. Hjelmeland, Ana Vazquez, Shannon M. Bailey, Jann N. Sarkaria, Corinne E. Griguer

**Affiliations:** 1 Department of Neurosurgery, University of Alabama at Birmingham, Birmingham, AL 35294 USA; 2 Department of Radiation Oncology, University of Iowa, Iowa City, IA 52242 USA; 3 Department of Pediatrics, University of Alabama at Birmingham, Birmingham, AL 35294 USA; 4 Department of Cell, Developmental, and Integrative Biology, University of Alabama at Birmingham, Birmingham, AL 35294 USA; 5 Department of Epidemiology and Biostatistics, Michigan State University, East Lansing, MI 48823 USA; 6 Institute for Quantitative Health Science and Engineering, Michigan State University, East Lansing, MI 48823 USA; 7 Department of Pathology, University of Alabama at Birmingham, Birmingham, AL 35294 USA; 8 Department of Radiation Oncology, Mayo Clinic, Rochester, MN 55902 USA; 9 Free Radical & Radiation Biology Program, 4210 Medical Education and Biomedical Research Facility (MERF), The University of Iowa, Iowa City, IA 52242-1181 USA

**Keywords:** Glioblastoma, Brain tumor, Chemoresistance, Temozolomide, IGF-1R, IGF2, IGFBP6

## Abstract

**Background:**

Glioblastomas (GBMs), the most common and most lethal of the primary brain tumors, are characterized by marked intra-tumor heterogeneity. Several studies have suggested that within these tumors a restricted population of chemoresistant glioma cells is responsible for recurrence. However, the gene expression patterns underlying chemoresistance are largely unknown. Numerous efforts have been made to block IGF-1R signaling pathway in GBM. However, those therapies have been repeatedly unsuccessful. This failure may not only be due to the complexity of IGF receptor signaling, but also due to complex cell-cell interactions in the tumor mass. We hypothesized that differential expression of proteins in the insulin-like growth factor (IGF) system underlie cell-specific differences in the resistance to temozolomide (TMZ) within GBM tumors.

**Methods:**

Expression of IGF-1R was analyzed in cell lines, patient-derived xenograft cell lines and human biopsies by cell surface proteomics, flow cytometry, immunofluorescence and quantitative real time polymerase chain reaction (qRT-PCR). Using gain-of-function and loss-of-function strategies, we dissected the molecular mechanism responsible for IGF-binding protein 6 (IGFBP6) tumor suppressor functions both in in vitro and in vivo. Site direct mutagenesis was used to study IGFBP6-IGF2 interactions.

**Results:**

We determined that in human glioma tissue, glioma cell lines, and patient-derived xenograft cell lines, treatment with TMZ enhances the expression of IGF1 receptor (IGF-1R) and IGF2 and decreases the expression of IGFBP6, which sequesters IGF2. Using chemoresistant and chemosensitive wild-type and transgenic glioma cells, we further found that a paracrine mechanism driven by IGFBP6 secreted from TMZ-sensitive cells abrogates the proliferation of IGF-1R-expressing TMZ-resistant cells in vitro and in vivo. In mice bearing intracranial human glioma xenografts, overexpression of IGFBP6 in TMZ-resistant cells increased survival. Finally, elevated expression of IGF-1R and IGF2 in gliomas associated with poor patient survival and tumor expression levels of IGFBP6 directly correlated with overall survival time in patients with GBM.

**Conclusions:**

Our findings support the view that proliferation of chemoresistant tumor cells is controlled within the tumor mass by IGFBP6-producing tumor cells; however, TMZ treatment eliminates this population and enriches the TMZ-resistant cell populationleading to accelerated growth of the entire tumor mass.

## Background

Glioblastoma (GBM) is the most common form of brain tumor, with 5- and 10-year survival rates of only 4.5 and 2.7%, respectively [1]. The standard treatment for patients with primary GBM includes concomitant and adjuvant chemotherapy with temozolomide (TMZ). Although chemotherapy is commonly used as part of the treatment for GBM and can be advantageous for short periods, chemoresistance eventually develops, causing chemotherapy to fail. Indeed, chemoresistance is the most challenging problem in cancer treatment and is the main reason for chemotherapy failure, yet the molecular mechanisms involved in the development of chemoresistance are not well characterized.

Intra-tumor heterogeneity is a signature of GBM and is associated with prognosis and response to treatment [2]. However, neither the cell-specific genotypes within GBM tumors nor the consequences of these differential genotypes have been fully defined. The insulin-like growth factor (IGF) system has a crucial role in tumorigenesis, and dysregulation of individual proteins within this system have been linked to GBM. In general, activation of this signaling pathway leads to increased mitogenesis, cell cycle progression, and protection against different apoptotic stresses [3]. The IGF system consists of soluble ligands (IGF1 and IGF2), cell surface transmembrane receptors (IGF1 receptor (IGF-1R) and IGF2 receptor (IGF-2R), and soluble binding proteins (IGFBP1–6) [4]. IGF-1R is critically involved in cell transformation and in the maintenance of the transformed phenotype by mediating the action of IGF1, IGF2, and insulin. Binding of ligand to IGF-1R activates the tyrosine kinase activity of the receptor, which leads to activation of the AKT signaling pathway [3]. Most activities of IGF1 and IGF2 are mediated by IGF-1R, and these activities are regulated by IGFBP1–6 [5]. IGFBPs bind with high affinity to both IGF1 and IGF2, but not insulin, and modulate the activity of these ligands by sequestration. In addition to modulating the binding of IGF1 and IGF2 to IGFRs, IGFBPs transport IGF1 and IGF2 into circulation and control the localization of these ligands in specific tissues [6, 7]. IGFBP6 is a unique member of this family, as it has a 50- to 100-fold higher affinity for IGF2 over IGF1. The major function of IGFBP6 is to inhibit IGF2-induced proliferation, migration, and survival of cells [8].

A subset of human gliomas appears to express IGF1 and/or IGF2 [9], and evidence suggests that IGF2 contributes to the aggressive nature of some GBMs [10]. Furthermore, the expression of IGF-1R in GBM associates with shorter survival and poor response to TMZ [11]. Notably, IGFBP6 inhibits proliferation and tumor development in many cancer types, including neuroblastoma [12], colon [13], ovarian [14], prostate [15], and rhabdomyosarcoma [16]. However, the endogenous source of IGFBP6 remains unidentified. We hypothesized that the differential expression of IGF system proteins by cells within GBM tumors regulates intra-tumor variation in tumor cell aggression and response to treatment.

Our results provide evidence that GBM tumor heterogeneity is maintained by a paracrine signaling circuit involving the IGFBP6-IGF2-IGF-1R axis that abrogates proliferation of TMZ-resistant glioma cells. In this mechanism, IGFBP6 secreted by TMZ-sensitive cells inhibits the expansion/proliferation of TMZ-resistant cells by preventing IGF2-induced IGF-1R/AKT signaling.

## Methods

### Cell lines and plasmids

TMZ-sensitive U251 cells and TMZ-resistant (UTMZ) counterparts were grown in DMEM F12 medium plus L-glutamine supplemented with 7% heat-inactivated FBS, penicillin, and streptomycin. Cells were incubated at 37° in a humidified atmosphere containing 5% CO_2_. The TMZ-resistant cell line was obtained previously by progressive adaptation of the parental TMZ-sensitive cells (U251) to TMZ. Resistant clones were isolated at an early stage of drug treatment (2.5 μm TMZ) and then successively exposed them to incremental doses of TMZ (up to 1 mm) to yield clones that were operationally resistant to doses of TMZ that would uniformly kill all U251 parental cells; these clones were designated as UTMZ. UTMZ cells were maintained in 160 μm TMZ. For more than 50 passages, the resistance to TMZ was retained [17]. U251 cells showed increased expression of surface markers associated with mesenchymal subtype [18] and UTMZ cells display stem cell like phenotype [17, 19, 20].

To generate cell lines lacking IGFBP6, U251 cells were electroporated with one of five different IGFBP6-specific shRNAs or control scrambled shRNA (MISSION shRNA lentiviral vectors, Sigma). Stable cell lines were generated by selection with puromycin. UTMZ cells were electroporated with a CCSB-Broad LentiORF-IGFBP6 clone or empty vector (Dharmacon, Lafayette, CO). Stable cell lines were generated by selection with blasticidin.

Cell lines were grown continuously up to 10 passages, and then a new culture was started from frozen seed stocks. Cell lines were tested for mycoplasma contamination using the ATCC Mycoplasma Testing Service and authenticated by the ATCC authentication service utilizing short tandem repeat (STR) profiling.

To generate the wt-IGFBP6 (Swiss-Prot accession number P24592), a cDNA encoding full-length IGFBP6 cDNA was cloned into the pET-28a(+) expression vector (GenScript, Piscataway, NJ) using *Nde*I and *Xho*I restriction sites. The pET-28a(+) vector has an N-terminal His tag with a thrombin cleavage site. The mut-IGFBP6 was similarly generated, but on the basis of a previous report [21], we substituted Ala for the hydrophobic amino acids P93A/L94A/L97A/L98A before cloning. Expression of wt-IGFBP6 and mut-IGFBP6 was induced in BL21 (DE3) competent *E.coli* by treatment with 1 mM IPTG for 3 h at 37 °C. *E. coli* cell extracts were prepared with CelLytic B (Sigma) and subjected to affinity purification with Ni-NTA chromatography.

To generate recombinant IGF2 (Swiss-Prot accession number P01344.1), the cDNA encoding full-length IGF2 was cloned into the pET-51b(+) expression vector (GenScript) using *Sal*l and *Hind*III restriction sites. The vector has a cleavable N-terminal Strep-Tag II tag. A stop codon was introduced to eliminate the C-terminal 10xHis tag. Expression of IGF2 was induced in BL21 (DE3) competent *E.coli* by treatment with 1 mM IPTG for 3 h at 37 °C. *E. coli* cell extracts were prepared with CelLytic B (Sigma) and subjected to affinity purification with Strep-Tactin resin.

For all constructs, sequencing alignment results were confirmed to be consistent with the targeted insert sequences, and the flanking sequences of the cloning sites were correct. The sizes of inserted fragments were correct and free of unexpected bands that would suggest contamination. The DNA appearance and quality of the mini-prep results indicated that samples were clear and free of contamination, with OD260/280 values from 1.8 to 2.0.

### GBM xenograft lines

Xenografts were generated from unique tumors derived from different patients, and were kindly provided by Dr. J. Sarkaria at the Mayo Clinic (Rochester, MN). This subset of the Mayo Clinic GBM xenograft lines has been widely used for basic and translational studies and extensively characterized. Prior authorization from the Mayo Institutional Review Board was obtained for the use of human tissue to establish the xenograft lines, and all patients consented to participation in research at the Mayo Clinic. Molecular genetic alterations and the corresponding patient tumor histopathologic classifications of the xenografts have been previously described [22, 23]. To allow definitive identification of the tumor lines, a molecular signature for each xenograft line has been defined using microsatellite analysis, and the signature of a specific tumor line can be compared to this baseline signature for authentication [23]. The xenografts are maintained by serial transplant in athymic nude mice, and authentication of the human lines is determined by STR profiling performed by the UAB Heflin Center for Genomic Science at UAB.

### Cell surface biotinylation and IGF-1R identification

The cell surface proteins of glioma cells were labeled using EZ-Link Sulfo-NHS-LC-Biotin (Thermo Fisher Scientific, #PI21338) as we previously described [24]. After lysis, biotin-labeled proteins were captured with streptavidin beads and digested in trypsin. Tryptic fragments were identified by tandem mass spectrometry (LTQ-FT; ThermoElectron), after the elimination of proteins found in control, non-biotinylated samples. Western blots were performed using HRP-conjugated streptavidin (Thermo Fisher Scientific, #ENN100, dilution 1:10,000) or an IGF-1R-specific antibody (rabbit monoclonal anti-IGF-I Receptor β, clone D23H3, Cell Signaling Technology, #9750, dilution 1:1000).

### Fluorescence microscopy

Glioma cells were plated at a density of 4 × 10^4^ cells per coverslip. After incubation for 24 h, cells were stained with mouse monoclonal anti-human IGF-IR fluorescein-conjugated antibody, clone #33255 (R&D Systems, #FAB391F, dilution 1:200) or mouse IgG1 fluorescein-conjugated isotype control antibody (R&D Systems, #IC002F, dilution 1:200). After staining, coverslips were mounted on slides with Fluoromount-G and imaged by fluorescence microscopy. Each experiment was performed in triplicate and assessed in a blinded manner.

### In silico analysis

Correlations between patient survival and IGF-1R gene expression were plotted using Kaplan-Meier survival curves with data from TCGA obtained using GlioVis [25]. From a total of 669 GBM patients included in the TCGA-GBM dataset, we selected for the present analysis 155 samples corresponding to all grade IV tumors and 142 samples corresponding to primary GBM from the TCGA-GBMLGG dataset. All biopsies were collected before chemotherapy. We used an optimal cutoff of 9.73 for Kaplan-Meier survival analysis. The optimal cutoff was obtained from GlioVis and calculated using the maximally selected rank statistics to determine the optimal cutoff for continuous variables, as provided in the ‘survminer’ package [25].

### Tissue specimens

Frozen glioma tissue specimens were obtained from the collection of clinical specimens in the University of Alabama at Birmingham (UAB) Brain Tumor Tissue Bank obtained from patients who underwent surgical treatment at UAB Hospital between January 2001 and November 2011. None of these patients received chemotherapy or radiotherapy before the surgery [26].

### FACS analysis

Xenograft lines GBM12, GBM22, GBM39, and GBM59 and the isogenic TMZ-resistant xenograft lines GBM12-TMZ, GBM22-TMZ, GBM39-TMZ, and GBM59-TMZ were obtained from Jann N. Sarkaria at the Mayo Clinic. The xenograft lines were passed through mice as subcutaneous tumors and dissociated using a gentleMACS Dissociator and Tumor Dissociation Kit (Miltenyi Biotec Inc., #130–095-929) according the manufacturer’s protocol.

Cells were treated with FcR Blocking Reagent (20 μl/10^7^ cells, Miltenyi Biotec Inc., #130–059-901) for 10 min at 4 °C. Cells were then stained with mouse monoclonal anti-human IGF-IR fluorescein-conjugated antibody, clone #33255 (dilution 10 μl/10^6^ cells) or mouse IgG1 fluorescein-conjugated isotype control antibody (10 μl/10^6^ cells). After incubation for 20 min in the dark on ice, cells were washed three times with FACS buffer (PBS + 2% FBS) and then analyzed by flow cytometry with a BD Accuri™ C6 Cytometer (Becton Dickinson Biosciences). The percentage of IGF-1R^+^ and IGF-1R^−^ cells was determined with FlowJo software from Tree Star.

### Quantitative RT- PCR

RNA was isolated using the RNAeasy mini kit (Qiagen, Germantown, MD) according manufacturer’s recommendations, and reverse transcribed using Primescript RT Master Mix (Takara/Clontech, Mountain View, CA). The cDNA samples were then used for real-time PCR using IGF2 (Hs03929076) and IGFBP6 (Hs00181853) Taqman FAM/MGB probe/primer sets (Life Technologies/Thermo Fisher Scientific). PCR was performed on the Qiagen Rotor-Gene Q 2-Plex Real Time PCR Cycler using Premix EX Taq 2X Master Mix (Takara/Clontech) with 10 ng of cDNA in duplex reactions containing primer-limited Euk 18S rRNA VIC/MGB probe/primer set (Applied Biosystems 4319413E), with two replicates for each sample. DNA was denatured for 2 min at 95 °C, followed by 40 cycles of 15 s at 95 °C and 30 s of annealing/extension at 60 °C. IGF2 and IGFBP6 expression levels were normalized to 18S RNA (Euk 18S rRNA).

### Human IGF2 and IGFBP6 immunoassays

IGF2 concentration in cell-conditioned medium was determined using the Quantikine ELISA Human IGF-II Immunoassay (R&D Systems, #DG200) according to the manufacturer’s recommendations. IGFBP6 concentration in cell-conditioned medium was determined using the Human IGFBP6 ELISA Kit (Abcam, #ab100544) according to the manufacturer’s recommendations.

### Western blot analysis

Cells were lysed in CelLytic M buffer (Sigma, #C2978) with the addition of Halt protease and phosphatase inhibitor cocktails (Thermo Fisher Scientific, #P178444, dilution 1:100) over 15 min at room temperature, then centrifuged for 10 min at 14,000×*g*, after which the insoluble debris was discarded. Cell lysates (20 μg of protein) were fractionated by 4–20% Mini-PROTEAN TGX Precast Protein Gels (Bio-Rad, #45610944) and transferred to Immobilon™-P PVDF Transfer Membranes (EMD Millipore). The membranes were blocked for 1 h and incubated overnight at 4 °C with the following primary antibodies: AKT (5G3) Mouse mAb #2966, IGF-I Receptor β (D23H3) XP Rabbit mAb #9750, phospho-IGF1 Receptor β (Tyr1135/1136) /Insulin Receptor β (Tyr1150/1151) (19H7) Rabbit mAb #3024, phospho-AKT (Ser473) (D9E) XP Rabbit mAb #4060, and Rab11 (D4F5) XP Rabbit mAb #5589 (all from Cell signaling at a dilution of 1:1000), and IGFBP6 antibody (C-20) (Santa Cruz, #sc-6007, dilution 1:500). The membranes were then incubated with secondary antibodies for 1 h at room temperature (goat anti-rabbit IgG (H + L) secondary antibody, HRP, #31460 and goat anti-mouse IgG (H + L) secondary antibody, HRP, #31430 (Thermo Fisher Scientific, dilution 1:10,000). The membranes were developed using Clarity Western ECL Substrate (Bio-Rad, #1705060S).

For Western blot ligand binding assays, wt-IGFBP6 and mut-IGFBP6 proteins were separated by 4–20% SDS-PAGE and transferred to PVDF membranes. Membranes were blocked for 1 h and incubated with 0.1 μg/ml of Strep-Tag II-IGF2 for 16 h at 4 °C. After incubation, the membranes were washed, blocked for 1 h, and incubated with biotinylated NWSHPQFEK Tag antibody (Genscript #A01737, dilution 1:1000). The blots were developed using streptavidin-HRP.

### Mouse models

We performed all surgical and experimental procedures and animal care in accordance and compliance with the policies approved by the UAB Institutional Animal Care and Use Committee (APN 20570). Intracranial gliomas were generated using 1 × 10^5^ human glioma cells suspended in 5% methylcellulose in serum-free medium. The cells were drawn into a 250-μl Hamilton gas-tight syringe mounted in a Chaney repeating dispenser and fitted with a 30G ½-inch needle with a calibrated depth of 2.5 mm from the middle of the bevel opening. Under an operating microscope, the fascia on the skull of the anesthetized mouse was scraped off and a 0.5-mm burr hole was made 2 mm to the right of the midline suture and 1 mm caudal to the coronal suture. The syringe was inserted into a Kopf stereotactic electrode clamp mounting bracket attached to an electrode manipulator (David Kopf Instruments; Tujinga, CA) mounted on a Kopf stereotactic frame electrode A-P zeroing bar (#1450). Each mouse was positioned on the stereotactic frame and the needle inserted to the depth marker in the right cerebral hemisphere. Approximately 90–120 s after injection of 5 μl, the needle was slowly withdrawn over the next 1 min. The burr hole was plugged with sterile bone wax and skin was closed with Tissumend surgical adhesive (Stryker Orthopedics, Kalamazoo, MI). The major endpoint in this study was animal survival; moribund animals that became unresponsive to mild external stimuli were euthanized, and the date was used as an estimate of the date of death. For flank tumors, 0.5 × 10^6^ cells were injected into the rear flank of 6-week-old nude mice (Envigo, Indianapolis, IN). Flank growth was monitored once every week with calipers to estimate tumor volume. The animals were euthanized on day 50 and the tumors were removed and tumor weight was determined.

### Statistical analysis

Data were evaluated using GraphPad software. All reported *p* values are for two-tail t tests, and *p* < 0.05 was considered to indicate statistical significance. Experiments were performed in triplicate and performed twice or more to verify the results. Data are shown as the mean ± S.D., with *p* < 0.05 (*), *p* < 0.01 (**), and *p* < 0.001(***).

## Results

### IGF-1R expression is increased in chemoresistant human glioma cells

**Fig. 1:**
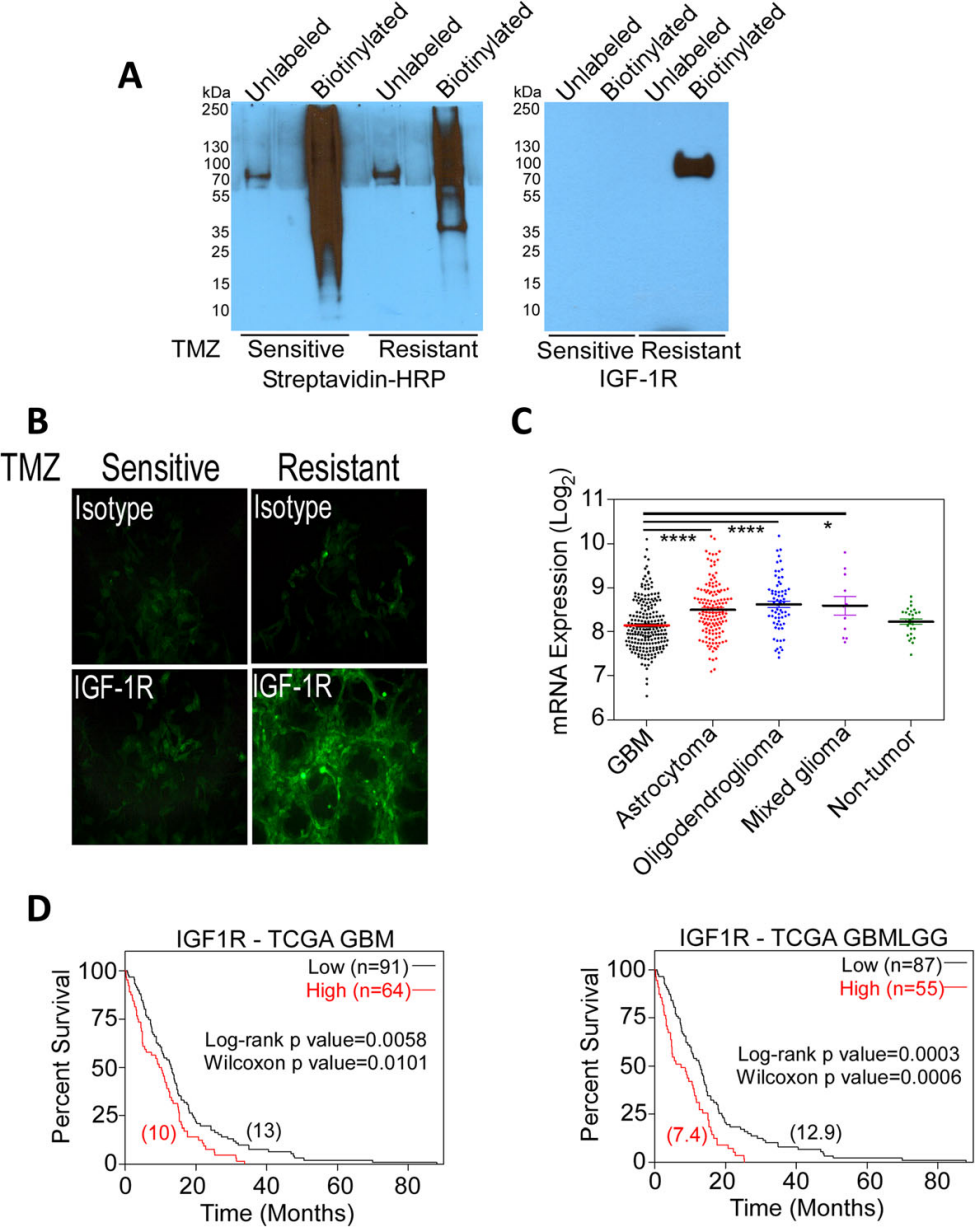
IGF-1R is preferentially expressed in TMZ-resistant glioma cells. **a** Cell surface biotinylated proteins, derived from lysed TMZ-sensitive (U251) and TMZ-resistant (UTMZ) glioma cells, were resolved on SDS/PAGE gels, and transferred to PVDF membranes. Proteins were detected by Western blot with HRP-streptavidin (left), and anti-IGF-1R antibody (right). **b** Visualization of the cell surface of non-fixed, live TMZ-sensitive U251 and TMZ-resistant UTMZ glioma cells with immunofluorescence using fluorescein-conjugated antibodies against IGF-1R (bottom panel) and isotype control (top panel). Magnification 400×. **c** Expression of IGF-1R in GBM and low-grade gliomas compared with non-tumor tissue. Median values are represented as lines in the scatter plot. *P < 0.05, ****P < 0.0001, ANOVA. **d** Expression of IGF-1R in GBM correlated with patient survival in the TCGA database (optimal cutoff 9.73): TCGA GBM dataset (left) and TCGA GBMLLG dataset (right). Numbers between brackets indicate median survival

To first determine whether IGF-Rs are differentially expressed by TMZ-sensitive and TMZ-resistant glioma cells, we biotinylated the extracellular domains of membrane proteins expressed by the isogenic TMZ-sensitive and TMZ-resistant human glioma cell lines U251 and UTMZ [17]. After pull-down with streptavidin beads, biotinylated proteins were detected by immunoblotting (Fig. 1a, left). Mass spectrometry and Western blot analysis of the protein fractions revealed that IGF-1R is expressed only by the TMZ-resistant UTMZ cell line (Fig. 1a, right). Immunofluorescence further showed that, compared with TMZ-sensitive U251 cells, the TMZ-resistant UTMZ cells expressed high levels of IGF-1R on the cell surface (Fig. 1b). Cell surface labeling for the isotype control groups confirmed that background fluorescence staining was negligible (Fig. 1b).

To confirm the clinical relevance of IGF-1R expression, we interrogated The Cancer Genome Atlas (TCGA) data accessed via GlioVis [25]. We identified several annotated brain tumor data sets in which we could compare IGF-1R expression in specimens of different tumor grades or from patients with differential survival outcomes. IGF-1R was significantly upregulated in astrocytoma, oligodendroglioma, and mixed glioma, whereas IGF-1R expression in glioma samples overall was lower and comparable to that in non-tumor tissues (Fig. 1c). However, elevated expression of IGF-1R within tumor samples from patients with GBM correlated with poor outcome (Fig. 1d).

**Fig. 2:**
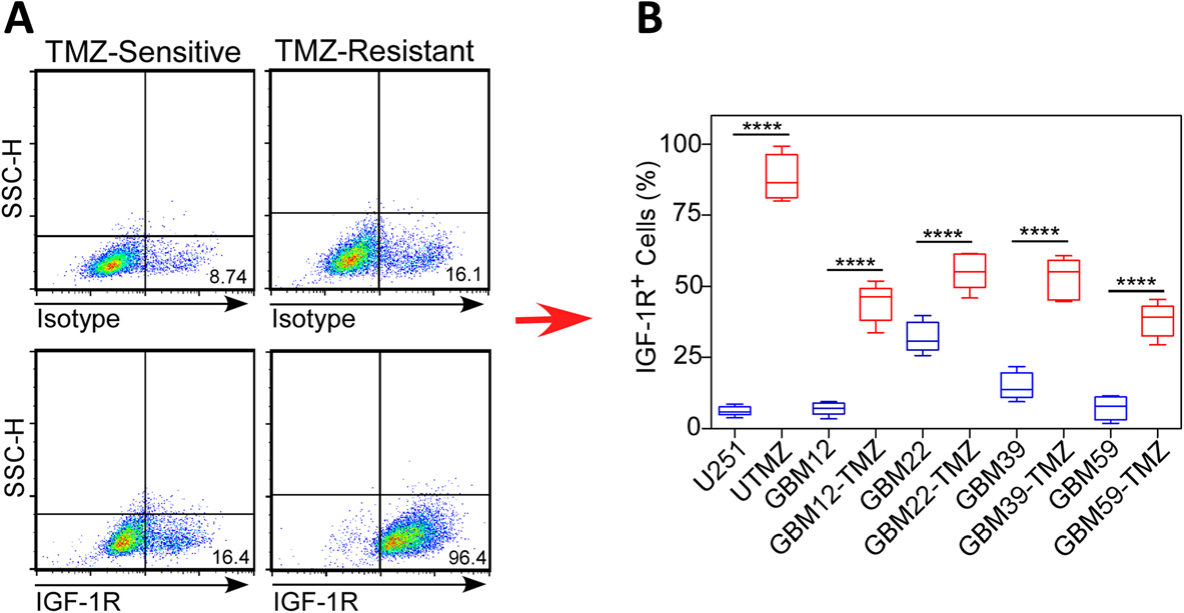
The expression of IGF-1R is upregulated in TMZ-resistant GBM xenograft lines. **a** Representative histograms showing the relative distribution of IGF-1R^−^ and IGF-1R^+^ cells in TMZ-sensitive (left) and TMZ-resistant (right) GBM xenograft lines. **b** Relative levels of IGF-1R expressed on the cell surface of TMZ-sensitive and TMZ-resistant GBM xenograft lines. ****P < 0.0001, two-tailed, t-test

Because protein expression under cell culture conditions does not always correlate with the in vivo condition, we extended our studies to a panel of serially transplantable GBM xenograft lines established by direct subcutaneous injection of patient tumor tissue in the flanks of nude mice. Using four GBM xenograft lines (GBM12, GBM22, GBM39, and GBM59), we developed corresponding TMZ-resistant models by subjecting mice with established flank tumors to successively higher doses of TMZ [22, 23]. We then assessed the cell surface expression of IGF-1R in the TMZ-resistant and TMZ-sensitive xenograft cells by FACS analysis. Representative histograms showing the distribution of IGF-1R^−^ and IGF-1R^+^ cells are shown in Fig. 2a. Figure 2b shows that IGF-1R expression was considerably higher in cells derived from TMZ-resistant xenografts than in cells derived from the corresponding TMZ-sensitive xenografts. Specifically, the percentage of IGF-1R^+^ cells in chemosensitive tumors ranged from 6.94% ± 0.90% (GBM12) to 31.91% ± 2.14% (GBM22), but after TMZ treatment to establish TMZ resistance, the percentage of IGF-1R^+^ cells ranged from 44.32% ± 2.70% (GBM12-TMZ) to 55.02% ± 2.49% (GBM22-TMZ) (Fig. 2b). These results suggest that an IGF-1R^+^ cell population is present in heterogeneous human GBM tumors and can be further enriched by TMZ treatment.

### TMZ-sensitive and TMZ-resistant glioma cells secrete different growth factors

**Fig. 3:**
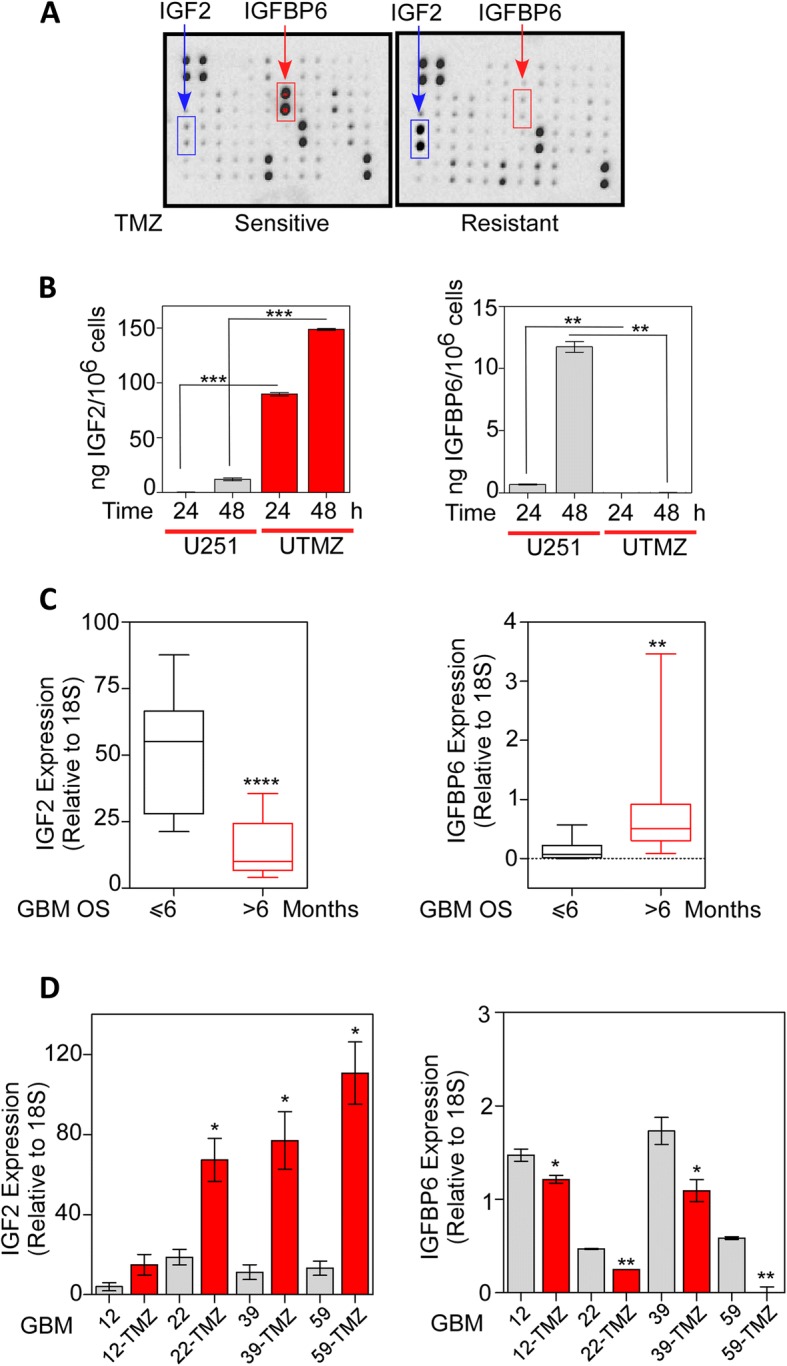
IGFBP6 and IGF2 are differentially secreted by TMZ-sensitive and TMZ-resistant glioma cells. **a** Determination of IGF2 and IGFBP6 expression in conditioned medium of TMZ-sensitive (U251, left) and TMZ-resistant (UTMZ, right) glioma cells using a human growth factor antibody array. **b** Quantitative analysis of IGF2 (left) and IGFBP6 (right) levels in culture medium of U251 and UTMZ glioma cells determined by ELISA. **c** Relative expression of IGF2 (left) and IGFBP6 (right) mRNA in tumor samples from GBM patients with OS less than or greater than 6 months. **d** Relative expression of IGF2 (left) and IGFBP6 (right) mRNA in TMZ-sensitive and TMZ-resistant GBM xenograft lines. mRNA expression was determined by qRT-PCR and normalized to 18S. *P < 0.05, **P < 0.001, ****P < 0.0001. Two-tailed, t-test

We next assessed the secretion of growth factors produced by TMZ-sensitive and TMZ-resistant glioma cells using a human growth factor antibody array to qualitatively detect 41 human cytokines and growth factors in conditioned medium of cultured cells. IGF2 was significantly upregulated in the conditioned medium of TMZ-resistant UTMZ cells compared with that of TMZ-sensitive U251 cells. On the other hand, the TMZ-sensitive cells secreted high levels of IGFBP6, whereas IGFBP6 was almost undetectable in the supernatants of the TMZ-resistant glioma cells (Fig. 3a). ELISA analysis of conditioned medium from both cell lines further showed that IGF2 accumulated in UTMZ-derived conditioned medium in a time-dependent manner but remained only marginally detectable over time in U251-derived conditioned medium (Fig. 3b, left). Conversely, high levels of IGFBP6 accumulated over time in U251-derived conditioned medium, but IGFBP6 was not detected in UTMZ-derived conditioned medium (Fig. 3b, right).

We next investigated the relationship between tumor IGF2 expression and the survival of patients diagnosed with primary GBM. IGF2 mRNA expression, detected by quantitative RT-PCR (qRT-PCR), was significantly upregulated in tumors from GBM patients with overall survival (OS) ≤6 months compared with tumors from patients with OS > 6 months (Fig. 3c, left). Conversely, higher expression of IGFBP6 was detected in tumors from patients with OS > 6 months (Fig. 3c, right).

Finally, we compared by qRT-PCR the expression of IGF2 and IGFBP6 mRNA in pairs of TMZ-sensitive and TMZ-resistant xenografts. IGF2 mRNA levels were 3.6- to 8.4-fold higher in the TMZ-resistant xenografts (Fig. 3d, left), whereas IGFBP6 mRNA levels were 1.2- to 20.0-fold higher in TMZ-sensitive xenografts (Fig. 3d, right).

These results revealed that IGF2 is significantly upregulated in the tumors of patients with TMZ-resistant GBM and low OS, whereas IGFBP6 expression is upregulated in the tumors of patients with TMZ-sensitive GBM and longer OS.

### IGFBP6 prevents IGF-2-mediated phosphorylation of IGF-1R in TMZ-resistant glioma cells

**Fig. 4:**
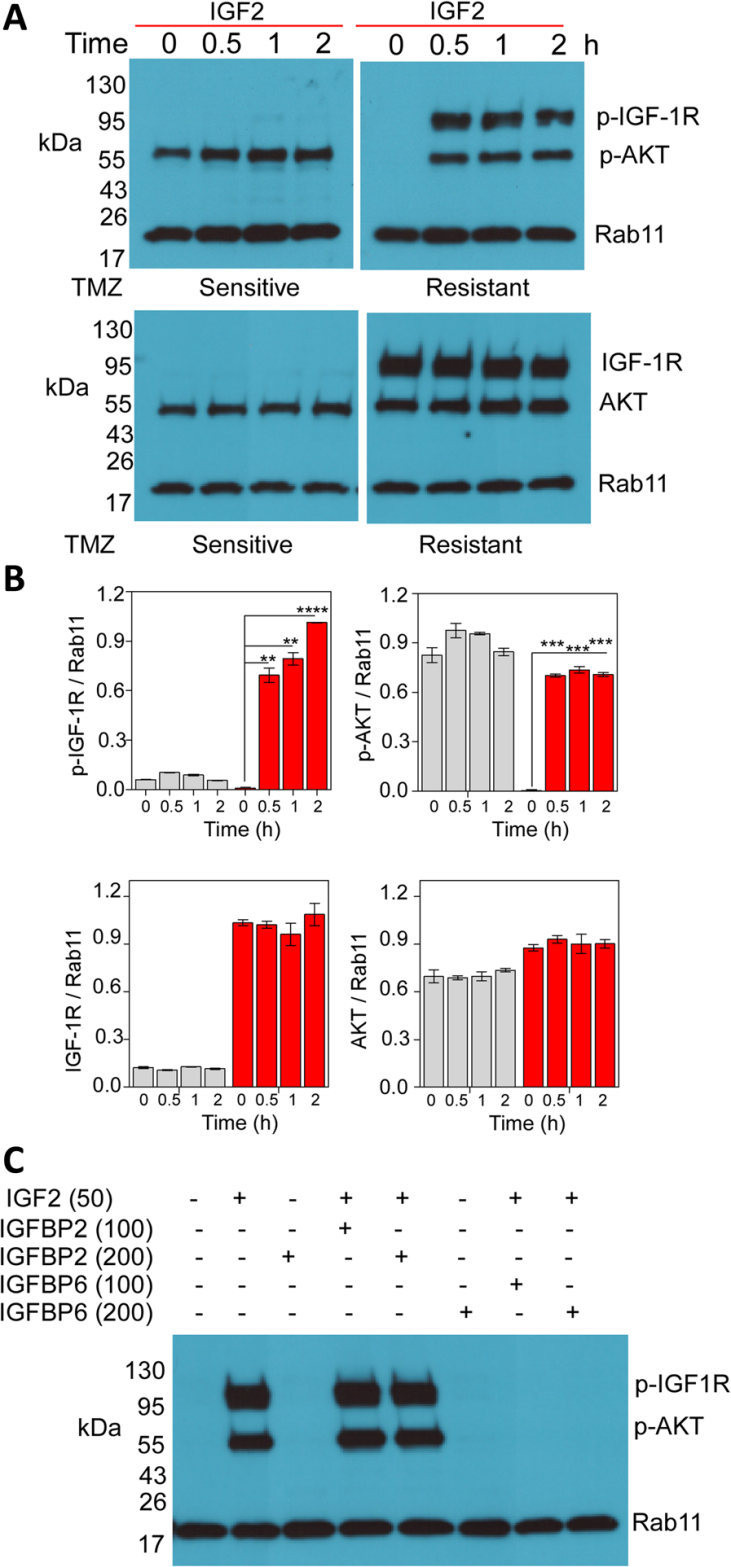
Activation of IGF-1R is abrogated after in vitro treatment with IGFBP6. **a** TMZ-sensitive (U251) and TMZ-resistant (UTMZ) cells were cultured in serum-free medium (SFM) for 16 h, then stimulated by IGF2 (50 ng/ml) for the indicated time. Cell lysates were subjected to Western blot analysis to determine the phosphorylation of IGF-1R and AKT (top panel) and total IGF-1R and AKT (bottom panel) in TMZ-resistant cells. AKT was constitutively activated in TMZ-sensitive cells. Representative blots are shown. **b** Quantitative analysis of protein expression levels from blots shown in (**a**). Bars represent the average from triplicate determinations from at least three independent experiments. **c** TMZ-resistant cells were starved in SFM overnight and then stimulated with IGF2 (50 ng/ml) in the presence or absence of recombinant IGFBP6 (100 or 200 ng/ml). Representative Western blots showing that IGFBP6 (but not IGFBP2) abrogated the IGF2-dependent phosphorylation of IGF-1R and AKT. Expression of Rab11 is shown as the loading control

IGF-1R activates the intracellular AKT signaling pathway. Therefore, we tested the effect of IGF2 on the phosphorylation of IGF-1R and AKT in TMZ-sensitive and TMZ-resistant glioma cells. In serum-starved TMZ-resistant UTMZ cells, treatment with IGF2 promoted the phosphorylation of IGF-1R (Tyr1135/1136) and AKT (Ser473). Robust phosphorylation of IGF-1R and AKT was detected within 0.5 h of treatment and lasted at least 2 h. In contrast, TMZ-sensitive U251 cells did not respond in this manner to IGF2 stimulation at any of the time points tested. Interestingly, however, TMZ-sensitive U251 cells displayed constitutive phosphorylation of AKT (Fig. 4a). We observe a slight increase in AKT phosphorylation in these cells after IGF2 stimulation (Fig. 4b) suggesting either that AKT activation occurs by distinct mechanism/s like transactivation of alternative receptors. We cannot also exclude the possibility that a different subcellular distribution or low levels of IGF-1R are present and responsible of AKT activation in chemosensitive cells. Thus, IGF2 secreted by TMZ-resistant glioma cells appears to function in an autocrine manner, phosphorylating IGF-R1 expressed by the TMZ-resistant cells.

Since IGFBPs block IGF activity, we next tested the effects of recombinant IGFBP6 on IGF2-mediated activation of IGF-1R in the TMZ-resistant UTMZ cells. Addition of IGFBP6 to the culture medium completely abrogated the IGF2-induced phosphorylation of IGF-1R and AKT. This effect may be specific for IGFBP6 since the addition of IGFBP2 at the same concentration did not block the phosphorylation of either IGF-1R or AKT (Fig. 4c).

**Fig. 5:**
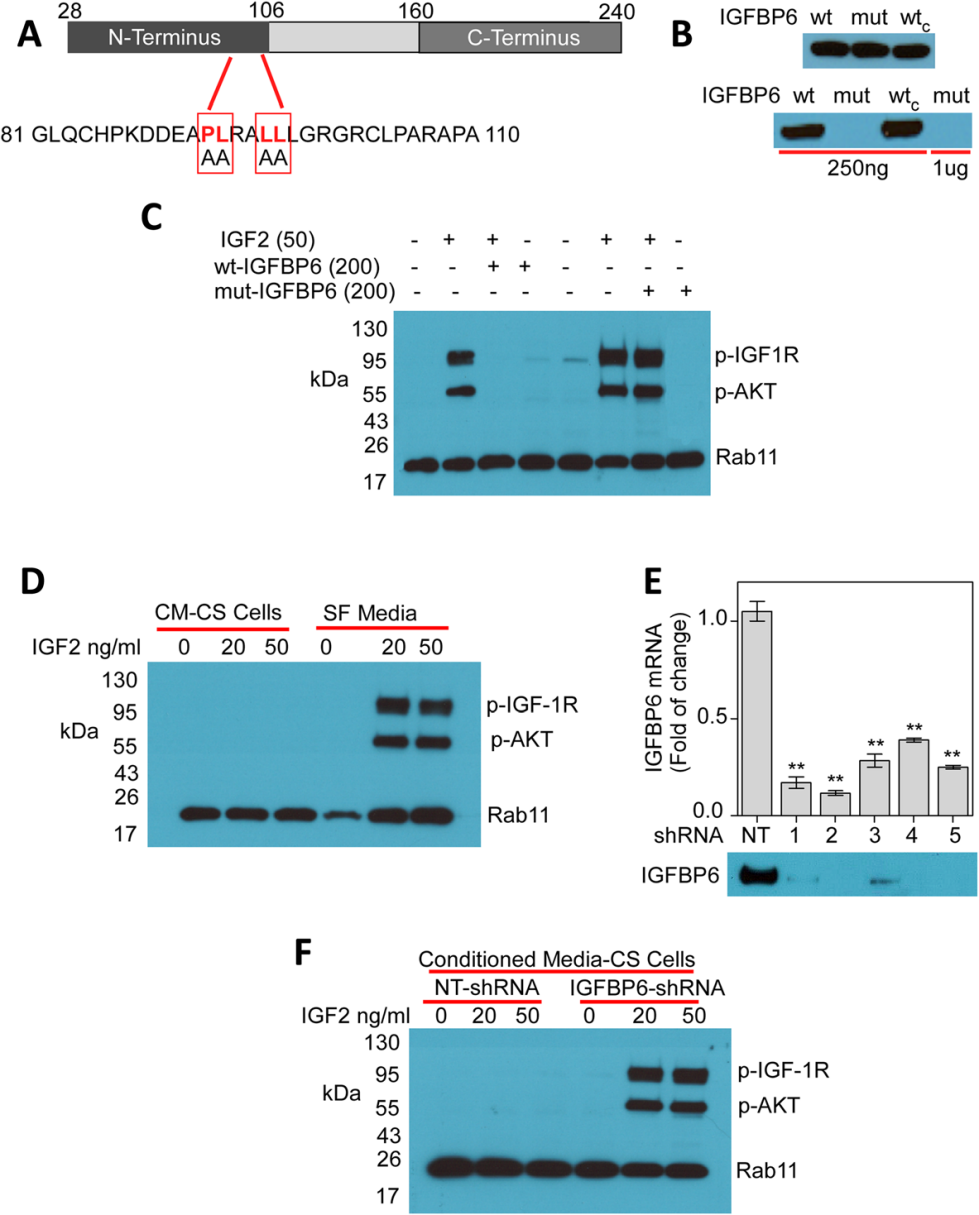
Abrogation of IGF2-mediated phosphorylation of IGF-1R is mediated by IGFBP6 binding and sequestration of IGF2. **a** Schematic representation of human IGFBP6 (Swiss-Prot, P24592) showing mutated sites. Amino acids mutated to Ala are shown in red. **b** Western blot analysis of recombinant IGFBP6 proteins (top panel). wt, wt-IGFBP6; mut, mut-IGFBP6; wt_c_, commercial wt-IGFBP6. Western blot ligand binding analysis (bottom panel). Wt-IGFBP6 (250 ng) and mut-IGFBP6 (250 ng or 1 μg) proteins were separated on 4–20% gels and transferred to PVDF membranes, incubated with Strep-Tag II-IGF2 and then developed with NWSHPQFEK Tag antibody. **c** Representative Western blot showing that wt-IGFBP6 (but not mut-IGFBP6) abrogates the IGF2-dependent phosphorylation of IGF-1R and AKT in TMZ-resistant cells. UTMZ cells were cultured in SFM for 16 h and then stimulated with IGF2 (50 ng/ml) in the presence or absence of recombinant wt-IGFBP6 or mut-IGFBP6 (200 ng/ml). **d** Representative Western blot showing that conditioned medium from chemosensitive cells abrogates the IGF2-dependent phosphorylation of IGF-1R and AKT in chemoresistant cells. UTMZ cells were cultured in SFM overnight and then stimulated with IGF2 at the indicated doses in the presence of conditioned medium from U251 cells. Expression of Rab11 is shown as the loading control. **e** Quantitative analysis (top) of extracellular IGFBP6 in conditioned medium of UTMZ cells stably transfected with one of five IGFBP6-shRNA constructs or a non-targeted control shRNA construct. Extracellular IGFBP6 was detected by Western blot (bottom) after 48 h of culture in SFM. Data are presented as the mean ± SEM of three independent experiments (*P < 0.05, **P < 0.001). **f** Representative Western blot showing that abrogation of the IGF2-dependent phosphorylation events requires the binding and sequestration of IGF2 by IGFBP6. UTMZ cells were cultured in SFM overnight and then stimulated with IGF2 at the indicated doses in the presence of conditioned medium from TMZ-sensitive U251 cells transfected with control shRNA or IGFBP6-shRNA constructs. Expression of Rab11 is shown as the loading control. CM, conditioned medium; CS, TMZ-sensitive cells; NT, non-targeted

To determine whether the ability of IGFBP6 to inhibit IGF2-induced phosphorylation of IGF-1R/AKT is mediated by specific binding and sequestration of IGF2, we generated a recombinant IGFBP6 mutant that is unable to bind IGF2 (mut-IGFBP6) by substituting Ala for four hydrophobic amino acids, P93A/L94A/L97A/L98A (Fig. 5a). In a Western blot binding assay, mut-IGFBP6 did not bind IGF2, even at a concentration four-fold higher than that at which a recombinant wt-IGFBP6 generated in our laboratory and a commercially available wt-IGFBP6 protein readily bound the growth factor (Fig. 5b). When added to the culture medium of TMZ-resistant UTMZ cells, mut-IGFBP6 did not abrogate the IGF2-dependent phosphorylation of IGF-1R and AKT, indicating that the specific binding and sequestration of IGF2 is required for these effects (Fig. 5c).

Since TMZ-sensitive glioma cells produce IGFBP6, we evaluated the effects of conditioned medium from TMZ-sensitive U251 cells on IGF2-dependent phosphorylation of IGF-1R and AKT expressed by TMZ-resistant UTMZ glioma cells. As controls, we generated clones of U251 cells in which IGFBP6 expression was downregulated by stable expression of IGFBP6-specific shRNAs (Fig. 5d). Exposure of the UTMZ cells to IGF2 in the presence of conditioned medium from the U251 cells did not lead to phosphorylation of IGF-1R or AKT (Fig. 5e). However, phosphorylation of IGF-1R and AKT occurred when UTMZ cells were exposed to IGF2 in the presence of conditioned medium from IGFBP6-depleted U251 cells (Fig. 5f). Taken together, these results indicate that IGFBP6 produced by TMZ-sensitive glioma cells can modulate IGF-1R/AKT signaling in TMZ-resistant cells.

### TMZ-sensitive glioma cell-secreted IGFBP6 inhibits proliferation of TMZ-resistant glioma cells

**Fig. 6:**
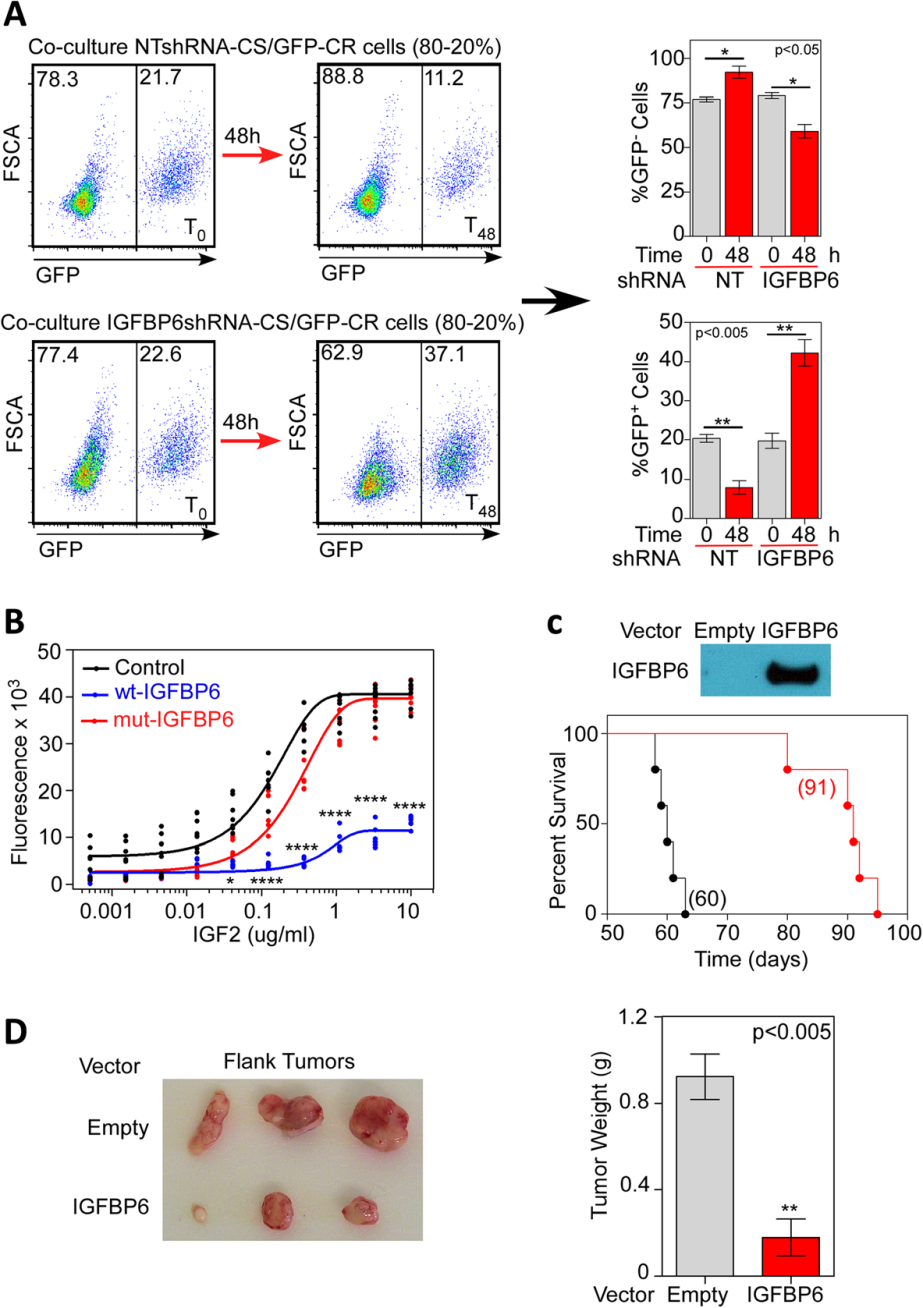
IGFBP6 abrogates proliferation of TMZ-resistant cells in vitro and in vivo. **a** GFP-labelled TMZ-resistant UTMZ cells were co-cultured with unlabeled TMZ-sensitive U251 cells transfected with non-targeted shRNA (control) or IGFBP6-shRNA (20%:80% ratio). After 48 h of co-culture the percentage of each cell population was determined by flow cytometry. Left: representative histograms show the distribution of GFP^+^ and GFP^−^ cells. Right: the percentage of GFP^−^ cells (top graph) and GFP^+^ cells (bottom graph) quantified after 48 h of co-culture. Data are presented as the mean ± SEM of three independent experiments (* *P* < 0.05, **P < 0.001). **b** UTMZ cells were plated at equal densities in 96-well plates and exposed to the indicated doses of wt-IGFBP6 or mut-IGFBP6. After 96 h, cell number was measured by CyQUANT Cell Proliferation Assays Kit. Results represent the mean of four independent experiments, with each treatment performed in quadruplicate within each experiment. (*P < 0.05, **P < 0.001, ****P < 0.0001). **c** UTMZ cells were transfected to stably overexpress IGFBP6. The representative Western blot shows the expression of IGFBP6 protein in the extracellular medium after 48 h of culture in SFM (top panel). Empty vector-transfected or IGFBP6-transfected UTMZ cells (1 × 10^5^) were intracranially implanted in athymic nude mice (*n* = 5). A significant improvement in survival was observed in mice bearing IGFBP6-transfected cells (Log-rank test, **P = 0.0018). Numbers in parentheses indicate median survival. **d** Representative images of tumors from athymic nude mice inoculated in the flank with empty vector-transfected or IGFBP6-transfected UTMZ cells (0.5 × 10^6^). Tumors were excised 50 days after inoculation (left panel). Comparison of tumor weights upon excision (right panel). (**P < 0.001). CR, TMZ-resistant; NT, non-targeted

We also examined the functional effect of TMZ-sensitive glioma cell-secreted IGFBP6 on TMZ-resistant cells. In co-culture assays, TMZ-sensitive U251 cells treated with control shRNA significantly inhibited the growth of GFP-expressing, TMZ-resistant UTMZ cells, whereas U251 cells treated with IGFBP6-shRNA did not. Specifically, when the GFP-expressing UTMZ cells were co-cultured with control shRNA-treated U251 cells, the percentage of GFP^+^ cells decreased from 20.00% ± 0.97% to 9.25% ± 1.26% after 48 h; when co-cultured with IGFBP6-depleted U251 cells, however, the percentage of GFP^+^ cells increased from 20.74% ± 1.05% to 42.22% ± 3.40% after 48 h (Fig. 6a).

Moreover, treatment of TMZ-resistant UTMZ cells with IGF2 caused a dose-dependent increase in cell growth, but simultaneous treatment with wt-IGFBP6 protein attenuated this effect. The addition of mut-IGFBP6 to the extracellular medium had no inhibitory effect on cell proliferation (Fig. 6b).

Finally, to evaluate the role of IGFBP6 in the progression of glioma cells in vivo, we engineered TMZ-resistant UTMZ glioma cells that stably overexpress and secrete IGFBP6 protein (Fig. 6c, top). Tumors were induced in nude mice by intracranial injection of 1 × 10^5^ empty vector- or IGFBP6-transfected TMZ-resistant cells per mouse. Overexpression of IGFBP6 significantly prolonged survival in animals bearing the orthotopic TMZ-resistant cells (Fig. 6c, bottom). When UTMZ cells were injected into the flank of nude mice, the mean tumor weight was significantly lower (0.18 ± 0.09 g vs 0.92 ± 0.1 g) in the group that received IGFBP6-transfected UTMZ cells than in the control group that received empty vector-transfected UTMZ cells (Fig. 6d). These results strongly suggest that IGFBP6 secreted by TMZ-sensitive glioma cells controls the proliferation of TMZ-resistant glioma cells in vitro and in vivo.

## Discussion

**Fig. 7:**
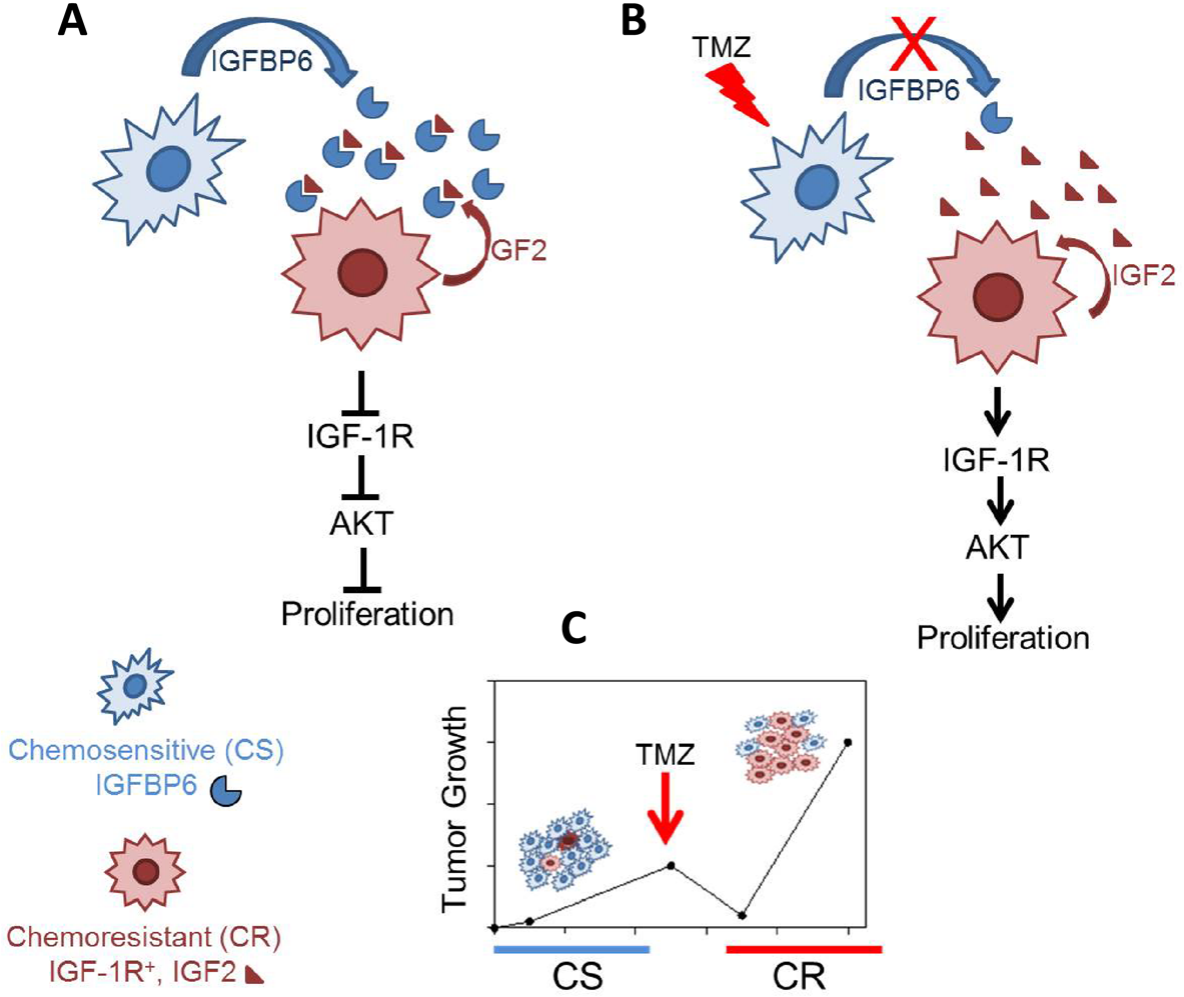
Model of paracrine-mediated regulation of chemoresistant cell proliferation in the context of glioma tumor heterogeneity. **a** TMZ-sensitive cells (blue) secrete IGFBP6, which binds and sequesters IGF2 produced by TMZ-resistant cells (red). As a consequence, IGF-1R and AKT signaling is not activated by IGF2, and proliferation of TMZ-resistant cells is suppressed. After TMZ treatment (**b** and **c**), the population of IGFBP6-producing, TMZ-sensitive cells is reduced, leaving extracellular IGF2 free and thus able to bind to and activate IGF-1R on the TMZ-resistant cells, thereby activating AKT signaling. Coordinate activation of IGF-1R and AKT enhances the proliferation of these chemoresistant glioma cells

While much attention has been placed on GBM heterogeneity with regard to genomic, transcriptomic, proteomic, and metabolomic characteristics of individual cells [27], far less is known about how intratumoral interactions between heterogeneous cancer cells affect pathologic process and/or risk of treatment failure and tumor relapse. Here, we show that TMZ-resistant and TMZ-sensitive glioma cells co-exist within GBM tumors, and the interaction of these cells through the secretion of IGF2 and IGFBP6, respectively, regulates cell proliferation and growth of the tumor. While most studies in glioma have characterized individual components of the IGF/IGF-1R axis, our results show that intratumoral interactions between TMZ-resistant and TMZ-sensitive cells involve the IGF system and drive the fate of the tumor. On the basis of our findings, we propose that GBM tumor growth is characterized by a structured population with less aggressive clones (TMZ-sensitive-IGFBP6^+^-IFG-1R^−^) that block the expansion of more aggressive cells (TMZ-resistant-IGF2^+^IGF-1R^+^). Importantly, by selectively eliminating the TMZ-sensitive tumor cells, TMZ treatment destroys this structured organization and facilitates subsequent progression of the more aggressive and treatment-resistant cells (Fig. 7). This tumor cell organization resembles the paracrine signaling involved in bacterial quorum sensing, in which gene expression is regulated in response to fluctuations in cell-population density to shape bacterial communities [28]. The consequence of such paracrine regulation is that fate decisions may depend on the cellular neighborhood and may suit different needs, such as regulation of tumor biomass according to nutrient/oxygen availability. We have characterized a population of chemoresistant glioma cells in which AKT is activated through the binding of IGF2 to IGF-1R and indirectly repressed by IGFB6 secreted from chemosensitive glioma cells. To our knowledge this is the first characterization of such a population.

Our findings in cultured cells are in agreement with the expression profiles found in clinical samples and GBM xenograft lines. This novel paracrine mechanism provides a rational consequence for the IGF-1R heterogeneity often detected in human GBM. Experimental studies have shown that alterations in IGF system function can influence tumor proliferation and are associated with cancer risk in a wide range of human cancers [29]. The involvement of the IGF system in the development of GBM is also very well documented in cell cultures, animal models, and human epidemiological studies [30, 31]. As previously reported, IGF-1R expression has prognostic value, being negatively associated with cancer-related survival. Various studies have associated IGF-1R expression with poor outcome in patients with esophageal [32, 33], gastric [34], oral [35], or cervical [36] carcinomas. In GBM, IGF-1R was identified as an independent prognostic factor associated with shorter survival and a less favorable response to TMZ [11, 31, 37]. While most human glioma cell lines do not express IGFs, a subgroup of human gliomas were reported to express IGF1 and/or IGF2, indicating a possible autocrine component [9, 37–39]. Previous reports have shown that GBM overexpressing IGF2 are very aggressive and that overexpression of IGF2 is associated with highly proliferative subpopulations that display intense Ki-67 staining within heterogeneous tumors, suggesting that IGF2 promotes the development and growth of some GBMs [37, 40, 41].

IGFBPs also modulate important biological processes, including cell proliferation, survival, migration, senescence, autophagy, and angiogenesis. The actions of these proteins have been implicated in growth, metabolism, cancer, stem cell maintenance and differentiation, and immune regulation [42]. The overexpression of some IGFBP isoforms has been well documented in GBM [43–45], and it was recently demonstrated that IGFBP2 stimulates proliferation, invasion, and chemoresistance to TMZ in GBM cells via different signaling pathways [46–48]. Additionally, it has been demonstrated that the expression levels of PTEN and IGFBP2 are inversely correlated in human brain and prostate cancers, and it was suggested that IGFBP2 is a potential serum biomarker of PTEN status and PI3K/AKT pathway activation in patients with cancer [49]. IGFBP3 also has been shown to facilitate GBM tumor cell proliferation, invasion, and migration through the regulation of STAT-1 signaling [50]. Furthermore, it is increasingly accepted that IGFBP6 is involved in abrogating the proliferation, migration, and survival of cancer cells by inhibiting IGF2 [6, 51]. This phenomenon has been observed in many types of cancer, including neuroblastoma [12], colon [13], and ovarian [14]; however, the endogenous source of IGFBP6 was not previously known. Furthermore, although higher plasma IGFBP6 levels have been associated with a better prognosis for GBM patients [52, 53], the specific role of IGFBP6 in the development and progression of glioma was also unknown. Our results suggest that chemosensitive tumor cells are a source of IGFBP6, and secretion of this protein serves to inhibit GBM progression. Our results do not however address the origins of heterogeneity, and leave open the issue of how amplified IGF2^+^/IGF-1R^+^ and IGFBP6^+^ subpopulations arise during the establishment of a glioma.

## Conclusions

We propose a model of paracrine-mediated regulation of chemoresistant cell proliferation in the context of intra-tumor heterogeneity in GBM (Fig. 7). TMZ-sensitive cells secrete elevated levels of IGFBP6, which binds and sequesters IGF2. As a consequence, IGF-1R and AKT signaling is abrogated and proliferation of TMZ-resistant cells is suppressed. We speculate that the population of IGFBP6-producer cells is vulnerable to TMZ and thus is reduced during treatment, leaving the proliferation of chemoresistant cells unchecked.

Our observations suggest that IGF-1R/IGF2 can be considered a potential target for the treatment of chemoresistant gliomas. Currently available clinical data on anti-IGF-1R therapies have demonstrated that these targeting approaches can promote strong antitumor activities in several tumor types; however, the efficacy is likely to be limited to a small patient subset. A deeper and broader understanding of the biology of the IGF system in heterogeneous tumor cells and integration of biomarker studies in all clinical investigations will be critical for future research.

## Abbreviations

GBM: Glioblastoma
IGF1: Insulin-like growth factor 1
IGF-1R: Insulin-like growth factor 1 receptor
IGF2: Insulin-like growth factor 2
IGFBP2: Insulin-like growth factor-binding protein 2
IGFBP6: Insulin-like growth factor-binding protein 6
qRT-PCR: Quantitative reverse transcription-polymerase chain reaction
TCGA: The Cancer Genome Atlas
TMZ: Temozolomide
